# Malaria Not the Cause of Fever

**Published:** 1856-01

**Authors:** S. Littell

**Affiliations:** Surgeon to Wills Hospital for Diseases of the Eye and Limb


### MEDICAL EXAMINER.

NEW SERIES.—NO. CXXXIII.—JANUARY, 1856.

### ORIGINAL COMMUNICATIONS.

*Malaria not the Cause of Fever.* By S. LITTELL, M. D., Surgeon to Wills Hospital for Diseases of the Eye and Limb.

*Mr. Editor*,—In an article on the Etiology of Disease, published in a former number of the Examiner,* I intimated my belief that a part far too prominent and exclusive had been assigned to the agency of malarious exhalations, and briefly adverted to a few of the facts which had led me to the adoption of that opinion. I stated that the complaints supposed to be thus engendered, prevail at a period when electrical fluctuations are greatest, and the body debilitated and otherwise disordered by the protracted heat of summer, is most sensible to their impression; that they are often observed where there is no reason to suspect the operation of malaria; are notoriously reproduced by other causes after they have once occurred; and are promptly cured by means which eliminate no poison, but merely restore the lost tone of the system,—frequently indeed, by mental impressions alone. Convinced that the hypothesis, notwithstanding its general adoption, is unfounded in fact, and injurious in its influence on medical practice, I beg leave to offer a few additional considerations in support of the view which I have taken.

Were it true that intermittent and remittent fevers owe their

* July, 1854.

origin to paludal exhalations, or to malaria however generated, we should naturally expect to find them most prevalent when vegetable decomposition was greatest; but a moment's reflection will show that this is not so. In the Middle and Western States, and perhaps throughout our country, September is the sickliest of the autumnal months, and yet vegetable life still flourishes, often in almost undiminished vigor; the foliage preserves its verdure and freshness, and nature exhibits few symptoms of her approaching decay. The days, moreover, have become considerably shorter, the weather cooler, and it is evident therefore, not only that material for decomposition is not supplied in greater abundance, but that the causes which concur in that process are really less active than they were in the preceding months. Those seasons also which are characterized by an unusually late fall, vegetation being fostered by timely rains and long unchecked by frost, are precisely those in which autumnal fevers prevail more extensively, though of a milder type than under other circumstances. The year which has just passed may be adduced in illustration. Summer and autumn were both marked by cool, wet, and variable weather; the country perhaps never preserved its freshness to so late a period; and yet intermittent and remittent fevers were more than ordinarily frequent, not only in localities where they are usually met with, but also on elevated grounds celebrated for their salubrity, and even in the very heart of the city. Circumstances like these cannot be accounted for on the theory of malarious exhalation, but receive an easy solution from the agency of humidity in giving effect to electrical changes.

A striking instance of a district in which every circumstance of climate, soil, and atmosphere might be supposed to unite in the production on a grand scale of paludal exhalation, is mentioned by Dr. Hooker, in his Himalayan Journals.* The delta of the Soormah extends for a distance of eighty miles along the old bed of the Burrampooter, a river five miles broad, and forms an immense still and narrow sheet of deep clear water, called the Jheels. The area drained by the Soormah is scarcely raised above the level of the sea, and contains about ten thousand square miles. In the dry season the Jheels are marshy, but

* Vol. 2, p. 263.

during the rains, which are excessive on the neighboring mountains, they are entirely overflown; the water rising to within a few inches of the huts, which are built along the branches of the rivers which traverse it. The soil, sandy along the Burrampooter, is more muddy and clayey in the centre of the Jheels, with immense accumulations of vegetable matter in the marshes, consisting chiefly of decomposed grass roots and leaves. "The climate of Chattuc (one of the villages) is excessively damp and hot throughout the year, but though sunk amid interminable swamps, the place is perfectly healthy. Such, indeed, is the character of the climate throughout the Jheels, where fever and ague are rare; and though no situations can appear more malarious than Silpat and Cachar, they are in fact eminently salubrious. These facts admit of no explanation in the present state of our knowledge of endemic diseases. Much may be attributed to the great amount and purity of the water, the equability of the climate, the absence of forests, and of sudden changes from wet to dry; but such facts afford no satisfactory explanation." Undoubtedly they do not, on the supposition that malaria is necessarily concerned in the production of such complaints; but discard that hypothesis, and they receive obvious elucidation on the theory of their electrical origin. Humidity alone, when universal and constant, tends to preserve the electrical equilibrium, and the great extent of their surface gives to the Jheels the character of an inland sea; the circumstances mentioned by Dr. Hooker must render electrical vicissitudes slight and infrequent, and hence their exemption from the so-called miasmatic diseases.

There is no circumstance, indeed, connected with our autumnal fevers—their occasional epidemic prevalence, the influence of moisture, the comparative exemption of large cities, the agency of winds &c. &c.,—which cannot be more scientifically explained on the electrical, than on the miasmatic hypothesis; and the former has the additional advantage of substituting an adequate, existing, and recognizable cause, for one which rests on no certain foundation, eludes all chemical scrutiny, and is to a great extent, if not altogether, imaginary.

For the modus operandi of this principle in the production of disease generally, of which it is a prolific proximate cause, I may be permitted to refer to the paper to which allusion has just been made ; but it may not be superfluous to unfold in few words the rationale of its action in the present instance.

Besides the effect of heat in rarifying the atmosphere, and thus diminishing the absolute quantity of oxygen inhaled, it excites the brain, and through it the other organs, to increased action; which, when the stimulus is withdrawn, if not before, is followed by corresponding nervous exhaustion. This increased action may terminate in vascular irritation and inflammation, inducing diarrhœa, cholera, dysentery, yellow fever, &c. &c., or may leave the system in a state of mere general debility, with little more than a tendency to congestion, chiefly of the portal circle. In this condition of increased susceptibility and diminished power, electrical variations, which would have little influence in a vigorous state of health, become a frequent and potential cause of disease, and their operation is promoted by various meteorological circumstances, as cold, moisture, &c. The cerebral functions are impaired, innervation is lessened, vascular congestion takes place, and, reaction following, the usual febrile phenomena are developed, which assume an intermittent, remittent, or typhoid form, according to the intensity of the cause, or the degree of the pre-existing debility.

The objection to this view, that disturbances of the electrical equilibrium are of constant occurrence without being followed by such effects as are here attributed to them, is more specious than sound. It fails in not appreciating the consequences of the protracted heat of summer, which exhausting the nervous energy, leaves the system, in the early autumnal months, weak, susceptible, and predisposed to disease; and it is, moreover, not altogether true in fact, for intermittent and remittent fevers are observed, though of course much less frequently, at other seasons of the year. As winter approaches, the invigorating influence of cold is felt in the increase of nervous power; oxygen is breathed in greater abundance with a denser atmosphere ; reaction follows the previous depression ; and the predisposition changes to other forms of morbid action. It is to this circumstance, and not to the destruction by frost of malarious exhalation, that the cessation of our autumnal fevers should be ascribed. The grateful impression and remedial agency of cold artificially applied, in almost all febrile conditions, is matter of common observation.

By Dr. Currie, of Liverpool, the effusion of cold water was employed with signal success in the treatment of scarlet fever, and I was informed by the late Dr. Littell, of Ohio, that in an epidemic dysentery of unusual severity which prevailed in his neighborhood, he resorted to the same means, with similar restrictions, and with the most gratifying results.

I do not deny that the foul emanations arising from the decomposition of animal and vegetable matter, which vitiate the atmosphere of our large cities, are a concurrent cause of disease. They are often present in a degree sensibly offensive, and cannot be otherwise than extremely prejudicial to health. Such vitiation, and the impression of excessive and long continued heat—the latter being absolutely necessary to its production—are powerful predisposing causes of yellow fever, and only require an atmosphere negatively electrical to give full effect to their deleterious agency. The increased conducting property of the air when loaded with moisture, accounts for the first appearance of the disease in the immediate vicinity of the rivers, or of the sea, upon which places subject to it are situated.* Yellow fever, as I have elsewhere observed, is an inflammatory affection which expends its force generally and principally on the stomach and collatitious viscera, but may be complicated also with inflammation of other parts predisposed to increased action. It runs its course with great rapidity, and derives its fatality both from its involving vital organs, and from its being engrafted upon an exhausted state of the system. My personal experience of the malady has not been very extensive, but I well recollect one case, occurring many years ago in a tropical climate, which was arrested in limine by venesection to the extent of fifty ounces. Early and copious depletion by the lancet, to reduce vascular excitement and change the character of the circulating fluid; calomel in large and frequently repeated doses, to relieve visceral congestion; and, these objects accomplished, or in progress, the liberal exhibition of quinine, to restore impaired nervous power

* In the cholera which prevailed at Columbia in the summer of 1854, the wind blew for several days in a particular direction, wafting over the town the air of a marsh saturated with moisture and impregnated with animal effluvia. This disease everywhere evinces a preference for the great water-courses of a country.

and impart tone to the extreme vessels; is the practice which, guided by a discriminating judgment, would follow by rational deduction from the theoretical opinions which I have advocated. Bleeding, however, to be safe and effectual should be employed in its incipient stage; the delay of a few hours would probably make all the difference between life and death.

It is with no little satisfaction that I have lately seen these speculative notions fully confirmed, with singular coincidence of sentiment, in the practice of Dr. Thomas Neilson, of Trinidad. During a residence of more than forty years on that Island, Dr. N. has witnessed no less than six epidemics of yellow fever, and the following extracts from a statement published by him in the Dublin Tablet, for which I am indebted to the Inquirer of this city, can hardly be further abridged without impairing the impression it is calculated to make. He states as his opinion,

"That it is a fever of inflammatory type, running its course from twenty-four to seventy-hours, and it is not contagious, but depending upon a vitiated state of the atmosphere, it being in a negative state of electricity—that is to say, deficient in oxygen.

"On the 25th June, 1853, W. Purdie, Esq., botanist, a native of Scotland, residing at St. Ann's, aged 33, of a sanguineous temperament, sent for me at five, A. M., to see him, being taken ill during the night with severe headache, pain over the epigastric region and over the eyeballs, as also of the calves of the legs, with inclination to vomit. He had thirst, pulse upwards of 100; the tongue white and slimy. I bled him to forty ounces, when he fainted and broke out into a profuse perspiration; the blood of a tarry black color. I gave him ten grains of calomel mixed with sugar, and ordered five grains every two hours, to be given until my return at 10 A. M., at which period all his pains had returned in an aggravated form. I removed the bandage from his arm, and bled him to about 24 ounces, when he fainted again and perspired profusely. All the symptoms were alleviated during the day. The calomel did not act as I expected. At 5 P. M., when I returned, I ordered one drop of croton oil on sugar every hour until it acted. He took nothing during the night but thin sago from time to time. He passed a restless night, and at 6 P. M. of the 26th he complained of severe pain over the eyebrows, his skin dry and hot, his pulse upwards of ninety. As the calomel had not acted I then removed the bandage from his arm, and took twelve ounces of blood, when its appearance became more florid, and he fainted, and was bathed in perspiration, his pulse falling to 76; his skin became moist, no thirst, his liver acting, he taking from time to time a little thin sago. He slept that night, and was convalescent on the following morning, when I ordered him twelve grains of sulph. quinine, with ten grains Gregory's powder twice that day, giving orders that no animal food for five days to come should be given him. Five days from the day he was thus taken ill he left St. Ann's for one of the Five Islands, for change of air, and returned to St. Ann's ten days after, in perfect health.

On the 13th July, 1853, I was sent for to see his Excellency the Right Hon. Lord Harris,* who was taken ill during the night with fever, severe headache, and inclination to vomit, and pains all over him, his pulse upwards of 100. He was of a lymphatic temperament, a native of England, aged 44. I bled him when I saw him until he fainted. I gave him ten grains of calomel, with white sugar mixed; ordered nothing but thin sago or tous-les mois. At 10 A. M. I returned, found the headache returned, and the other symptoms in a very severe and aggravated form. He being governor of the colony I did not like to bear the entire responsibility, and requested his father-in-law, the Venerable Archdeacon Cummins, to call in Dr. Van Buren in attendance with me upon him. His symptoms becoming aggravated, we bled him to sixteen ounces, when he fainted; repeated the calomel, and ordered nothing but iced tous-les-mois or sago. During the night, his fever returning, we bled him every third hour, taking from three to five ounces of blood at each period, which broke the spasm, and he fainted; we gave him five grains of calomel with sugar every four hours. On the 14th his fever returned, and he was bled again from the arm to eight ounces, when he fainted, and perspired profusely. His medicine acted, and on the following morning he was convalescent. He then took during the day twelve grains of quinine, mixed with 24 grains Gregory's powder, in three doses, taking nothing during the period but thin sago or arrow-root."

Several other cases are related in detail. The whole number of patients under his care at St. Ann's, the residence of the Governor, was twenty-six, and in every instance he pursued, with slight variation, and uniform success, the same treatment. The following is the conclusion of the article.

"*I beg leave further to observe that any medical man using his lancet to take a certain quantity of blood without causing deliquium, or breaking the spasm upon the cutaneous vessels, I am of opinion he may as well shoot his patient, as it will run its course more rapidly and effectively.* He will see black vomit set in early from the attack.

"N. B.—An unfortunate circumstance for many families took place. The home government sent out Doctor Hector Gavin, as Health Inspector, to teach the resident prcatitioners how to treat yellow fever and cholera, and he so intimidated the junior practitioners from the use of the lancet *that nearly all their cases proved fatal.*

"In any case where black vomit had set in I put my trust in lime water, two table-spoonfuls to a quart of milk, to be drunk at pleasure, from which some cases have recovered; nothing else should be allowed. When the Fahrenheit thermometer ascends to 92 or 94 in the shade, with

* Governor of Madras.

the wind blowing west by north, and charged with moisture, you will have sporadic cases of yellow fever, with bilious remittent fevers, occurring frequently."

The testimony of his complete success, says the *Tablet*, is placed beyond all question by the following public address. Among the names we readily distinguish the most eminent of the Catholic and Episcopalian Clergy—the Rev. T. L. Richards, Protestant Rector of Port of Spain; the Very Rev. Thos. Smith, nephew of the late and ever-to-be-lamented Catholic Archbishop Smith, and others not forgotten in Maynooth. It is signed by the professions and leading merchants of the island, and those who desired to express gratitude for the preservation of their lives:

"TO THOMAS NEILSON, ESQ., M. D.

"*Trinidad, Jan.* 1*st*, 1855.

"We, the undersigned residents in Richmond and the other streets in Port of Spain, which were, during the prevailing late dreadful epidemic, entrusted to your professional care and supervision, desire to convey to you the expression of our grateful thanks for the deep interest which you continually evinced in our welfare. Not only do we willingly bear testimony to the *efficient manner in which you performed your important duties towards* all classes of persons, but we desire also to record our sense of gratitude for your many acts of liberality most generously exercised towards those who stood in need of assistance. In conveying to you these acknowledgments, we earnestly pray Almighty God that your valuable services may never again be required, either in this island or elsewhere, to check the violence of so dreadful a pestilence."

(Signed.)

A casual expression of Dr. Neilson, in the extract quoted above, would seem to intimate his belief in the identity of oxygen and electricity; an opinion certainly not warranted in the present development of chemical science. There is no doubt, however, that, from the rarefaction of the atmosphere, a smaller quantity of the former is taken into the lungs; in consequence of which the blood does not undergo due aeration, and all the organs suffer in a greater or less degree. Another efficacious source of exhaustion and irritation is the direct action upon the system, of excessive and protracted heat; and these causes thus combined, may, of themselves, give rise to various morbid phenomena. In this state of things, fluctuations in the quantity of an element which pervades all nature, and is strikingly analogous to, if not identical with, the nervous energy or force, cannot fail to have a very powerful influence over the cerebral functions. That they actually do exert a sensible power even in a state of health, exalting or depressing vitality, according as the electricity is in excess or otherwise, is matter of common experience and observation ; but in an exhausted condition of the nervous force the injurious impression resulting from the deficiency of that fluid, must necessarily be far more considerable; giving rise, with the auxiliary agencies just mentioned, to intermittent, remittent, bilious, and yellow fever ; and under predispositions induced by different meteorological states and combinations, to other maladies of epidemic prevalence, as well as to neuralgia, rheumatism, the phlegmasiæ, and various sporadic affections.

The subject open a wide field for pathological investigation, and will require on very many topics, a reconstruction of medical sentiment. Several complaints now attributed to contagion will be seen to spring from a cause which no isolation could evade ; demonstrating thus the inutility and folly, as well as the mischief of quarantine regulations. Not only malaria, but vaccination, except, in so far as it operates as an amulet,—inspiring confidence, and thereby preventing the depressing influence of fear*—and various other widely-adopted and long-cherished opinions, are destined to fall before the more rational theory which it involves ; while it cannot fail to exercise also a beneficial effect upon practice, in making the preservation and restoration of nervous energy a prominent object of regard, both in health and disease.

In the late expedition up the Niger, a river so often fatal to previous explorers, the health of the crew was preserved in a remarkable manner by the exhibition of quinine, morning and evening, with other hygienic precautions. Tonics are well known to act as preventives of our common autumnal fevers ; and it is worthy of experiment, whether this, or some article of similar cerebral impression, would not be found equally effectual as a prophylactic of yellow fever, and of other complaints, to the attacks of which physical debility or exhaustion has always been observed to give a strong predisposition. Whatever sustains and exalts the nervous power, must necessarily tend to avert disease, whether occurring sporadically or as an epidemic.

*Philadelphia, December*, 1855.

*It may be proper to say that in a matter like this, where human life and safety are concerned, I should think myself culpable in acting upon views opposed to those of the whole body of the Profession ; and therefore, notwithstanding my infidelity, continue to vaccinate with all the care of the most orthodox believer.

